# The influence of career calling on the work engagement of ideological and political theory teachers in colleges and universities: the chain mediating effect of organizational commitment and job satisfaction

**DOI:** 10.3389/fpsyg.2025.1666858

**Published:** 2026-01-20

**Authors:** Yulong Guo, Mengshi Zhang

**Affiliations:** 1 College of Marxism, Harbin Engineering University, Harbin, China; 2 School of Humanities and Law, Northeast Forestry University, Harbin, China

**Keywords:** career calling, chain mediating effect, job satisfaction, organizational commitment, work engagement

## Abstract

To explore the specific influence mechanism of career calling on the work engagement of ideological and political theory teachers in colleges and universities, based on Self-Determination Theory, Social Exchange Theory, Social Information Processing Model and previous relevant empirical research, this paper constructs a path model of organizational commitment and job satisfaction playing a mediating role between the teachers’ calling and work engagement. The analysis results of 1,428 survey data show that the teachers’ calling can not only directly and significantly affect their work engagement, but also indirectly and significantly affect their work engagement through the separate mediating role of organizational commitment and job satisfaction and the chain mediating role of the two. Based on these findings, several recommendations are provided to improve the work engagement level of ideological and political theory teachers in colleges and universities, including value guidance, organization reengineering, power activation and dynamic regulation.

## 1 Introduction

Work engagement is a positive, substantial, and work-related psychological state (Schaufeli et al., 2002a). Individuals who are engaged in work usually have three characteristics: one is vigor, which means that they have strong energy and good psychological resilience at work; the second is dedication, that is, to experience the sense of meaning, enthusiasm, encouragement, pride and challenge in work; the third is absorption, that is, the attention is highly focused on the current task field, triggering a significant compression effect of subjective time perception, and generating task disembedding resistance psychology (Schaufeli et al., 2002b). In China, ideological and political theory teachers in colleges and universities are the main force shouldering the responsibility of production and innovation of Marxist theoretical knowledge and driving the internalization and integration of Xi Jinping Thought on Socialism with Chinese Characteristics for a New Era into students’ thinking and hearts. Their work engagement is related to the implementation of the fundamental tasks of moral education in colleges and universities and the cultivation of socialist builders and successors. However, the existing empirical research only analyzes the mechanism of a few influencing factors such as job crafting and work meaning (Shang, 2022). In general, the influence mechanism of the work engagement of ideological and political teachers in colleges and universities is still a black box that needs to be explored urgently.

Career calling is a transcendent calling, which comes from beyond the self (Dik and Duffy, 2009). It requires people to assume a specific role in life in a way that is committed to showing or obtaining a sense of purpose and meaning, and to regard other-oriented values and goals as the main source of motivation. Career calling is the deepest (Hall and Chandler, 2005) and strongest (Bellah et al., 2007) source of meaning when individuals practice specific professional roles, and it is an important driving force for people to actively participate in work (Lin et al., 2025). As for teachers of ideological and political courses in colleges and universities, career calling provides a strong spiritual driving force for their work. Specifically, it stimulates their enthusiasm and initiative in teaching and educating students. This further enables them to wholeheartedly engage in the efforts to build the Chinese nation’s “dream team,” which dedicated to national development. With the dedication of a “ladder” (i.e., supporting others’ growth), they also uphold the ideal of national rejuvenation for the new generation of talents (Li and Guo, 2025). However, previous studies have not discussed the impact of career calling on the work engagement of ideological and political teachers in colleges and universities from an empirical perspective.

According to the basic psychological needs framework of Self-Determination Theory (S-DT) (Deci and Ryan, 2000), career calling can enhance organizational commitment by satisfying three fundamental psychological needs: autonomy, competence, and relatedness (DiRenzo et al., 2022). Under Social Exchange Theory (SET), organizational commitment serves as a bridge between the individual and the organization (Allen and Meyer, 1990), reinforcing work engagement through the pathway of emotional attachment to resource investment (Riketta, 2008). Therefore, organizational commitment plays an intermediary role between career calling and work engagement of ideological and political teachers in colleges and universities. From the perspective of motivational efficacy, career calling can continuously stimulate goal-directed behaviors and self-motivation mechanisms among university ideological and political education instructors. This effectively enhances positive work experiences through achievement feedback loops (Duffy et al., 2018; Zhang and Hirschi, 2021; Schultz et al., 2022). According to SET (Cropanzano and Mitchell, 2005), job satisfaction as a manifestation of positive emotional states can reinforce the reward motivation of university ideological and political education teachers. To maintain a positive exchange relationship with the organization, teachers proactively increase their work investment. Thus, organizational commitment and job satisfaction can mediate between career calling and work investment among university ideological and political education teachers. Moreover, according to the Social Information Processing (SIP) Model (Salancik and Pfeffer, 1978), university ideological and political education teachers with high organisational commitment tend to focus more on meaningful aspects within their work. This emphasis on meaning enhances their emotional investment in their role, thereby increasing job satisfaction (Wilson and Gilbert, 2005; Abu Orabi et al., 2024). However, previous research has not examined the separate and sequential mediating effects of organizational commitment and job satisfaction on the relationship between career calling and work engagement among university ideological and political education teachers.

To summarize, three critical research gaps remain in the existing literature. First, the antecedent mechanism of work engagement for college ideological and political teachers is insufficiently explored. Current studies only focus on limited factors (e.g., job crafting) and lack attention to career calling, a core spiritual driving force for this professional group. Second, the potential mediating roles of organizational commitment and job satisfaction between career calling and work engagement have not been empirically verified in the context of ideological and political teachers, despite theoretical support for their individual mediating effects. Third, the chain relationship between organizational commitment and job satisfaction (i.e., career calling to organizational commitment to job satisfaction to work engagement) has not been examined, leaving the multi-stage transmission path of career calling on work engagement unclear. These gaps limit our understanding of how to effectively enhance the work engagement of ideological and political teachers.

Against this backdrop, this study aims to fill the above research gaps by constructing a chain mediating model. Specifically, based on Self-Determination Theory, Social Exchange Theory, and the Social Information Processing Model, we empirically test: (1) whether career calling has a direct positive impact on the work engagement of college ideological and political teachers; (2) whether organizational commitment and job satisfaction play separate mediating roles between career calling and work engagement; and (3) whether organizational commitment and job satisfaction play a chain mediating role in the relationship between career calling and work engagement.

This study has both theoretical and practical significance, as elaborated below: First, it enriches the antecedent variable research on work engagement of college ideological and political teachers. By empirically verifying the direct positive impact of career calling on their work engagement, this study supplements the research on the spiritual driving factors of work engagement for this professional group with strong value attributes, breaking the limitation of existing studies focusing only on job crafting and work meaning. Second, it expands the application of chain mediating models in the field of ideological and political education. By confirming the separate and sequential mediating roles of organizational commitment and job satisfaction, this study clarifies the multi-stage transmission path of career calling on work engagement, which deepens the theoretical understanding of the black box between career calling and work engagement. Third, it strengthens the integration of multiple theories in specific professional contexts. For educational management departments and colleges and universities, the research results can provide targeted practical guidance for improving the work engagement of ideological and political teachers. This not only helps to stabilize the team of ideological and political teachers and stimulate their teaching enthusiasm but also provides practical guarantee for the implementation of the fundamental task of moral education and the cultivation of high-quality socialist builders and successors.

## 2 Literature review and development of hypotheses

### 2.1 Calling and work engagement

Career calling is defined as a transcendent summons originating beyond the self, which urges individuals to assume specific life roles in a manner committed to pursuing purpose and meaning, and to take other-oriented values and goals as the core source of motivation (Dik and Duffy, 2009). As a multi-dimensional construct, it typically includes three core components: transcendence links personal work to broader social values or spiritual pursuits, purposefulness possess clear goals related to professional practice, and proactivity takes initiative to align behaviors with calling expectations (Duffy et al., 2018; Hall and Chandler, 2005). Relevant studies have identified multiple factors interrelated with career calling. On the individual level, prosocial personality traits (such as empathy and altruism) and core self-evaluation positively predict the formation of calling (Kaminsky and Behrend, 2015; Lysova et al., 2019; Xu et al., 2023); on the contextual level, organizational value guidance (e.g., emphasizing the social significance of work) and mentor support can strengthen individuals’ perception of calling (Dik et al., 2009; Elangovan et al., 2010). For professional groups with strong value attributes, such as college ideological and political teachers, calling also shows a close connection with role identity: teachers who perceive a strong calling tend to integrate their professional roles into self-concept, regarding the spread of Marxist theory and the cultivation of socialist successors as an inherent part of self-realization. Empirically, career calling has been proven to be a key antecedent of positive work outcomes across various professions. In the education field, studies have shown that teachers with high calling perception exhibit higher teaching enthusiasm and professional persistence (Rawat and Nadavulakere, 2015; Dik et al., 2020); in the public service sector, calling mediates the relationship between public service motivation and work performance (Vandenabeele, 2008; Leisink and Steijn, 2009; Vogel, 2022; Wang and Liu, 2024).

Work engagement is a positive, fulfilling, and work-related psychological state characterized by three interrelated dimensions: vigor, dedication, and absorption (Schaufeli et al., 2002a,b). The formation of work engagement is influenced by the interaction of individual and contextual factors. Individual-level antecedents include autonomous motivation (Li et al., 2015; Moreira-Fontán et al., 2019), professional self-efficacy (Schaufeli et al., 2002a,b; Chan et al., 2017), and work meaning perception (Yasin Ghadi et al., 2013; Beukes and Botha, 2013); contextual factors mainly involve job resources (such as work autonomy, performance feedback, and social support) and organizational climate (e.g., supportive leadership and fair reward mechanisms) (Bakker and Demerouti, 2017; Bakker et al., 2023). Extensive empirical research has confirmed the positive consequences of work engagement. For teachers, high work engagement is associated with better teaching quality, higher student satisfaction, and stronger professional well-being (Rusu and Colomeischi, 2020; Wang, 2022; Shu, 2022); for colleges and universities, the work engagement of ideological and political teachers directly affects the implementation effect of moral education and the cultivation quality of talents (Shang, 2022).

According to S-DT, the degree of autonomous regulation of individual motivation, that is, the level of integration of behavior, core values and self-concept, is the core mechanism for predicting work engagement (Conway et al., 2015). S-DT divides autonomous regulation into four levels: extrinsic regulation, that is, behavior is driven by external rewards and punishments; introjected regulation, that is, behavior is driven by internal pressure, such as avoiding guilt; identified regulation, that is, the behavior is driven by the value of the identity target; integrated regulation, that is, the complete integration of behavior and self-concept, becomes the expression of “true self.” Among them, integrated regulation is the most autonomous motivation type, and individual behavior not only recognizes the target value, but also regards it as part of self-existence (Deci and Ryan, 2012). The calling is essentially a typical integrated adjustment motivation (Elangovan et al., 2010). Previous studies have pointed out that mission-driven individuals regard work as the carrier of life meaning (Duffy et al., 2018), and their work motivation comes from who I am and what I want to do (Zhu et al., 2025), that is, the deep unity of self-identity and work value. This integrated regulation motivation is different from the simple “identified regulation” that only recognizes the work value but does not integrate into the self-concept, and can stimulate continuous and active engagement behavior. Therefore, for ideological and political teachers in colleges and universities, the higher the sense of calling, the closer their work motivation is to the level of integrated adjustment, they will regard teaching and scientific research as the inevitable choice of self-realization, rather than task or responsibility (Li and Guo, 2025). This motivational trait will directly drive them to show higher initiative, persistence and emotional involvement in teaching design and scientific research investment, that is, higher work engagement level (Xie et al., 2016; Zhao et al., 2022; Xiang et al., 2024). Based on this, this study proposes the following hypothesis:

Hypothesis (H1): Calling can significantly positively affect the work engagement of ideological and political theory teachers in colleges and universities.

### 2.2 The mediating role of organizational commitment

Organizational commitment is a comprehensive reflection of individual recognition, participation and loyalty to the organization (Yousef, 2000). Its core is the deep connection between individual and organization in values, goals and emotions. Based on the basic psychological needs framework of S-DT (Deci and Ryan, 2000), this study proposes that career calling can enhance organizational commitment and drive work engagement by meeting the three basic psychological needs of individual autonomy, competence and belonging. Specifically, S-DT points out that human beings have three basic psychological needs: the need for autonomy, that is, behavior stems from internal will rather than external control; competency needs, that is, confidence in their own ability and growth experience; belonging needs, that is, to establish emotional connection with others/organizations (Ryan Richard and Deci, 2000). As a highly endogenous work motivation (Dik et al., 2020), the sense of calling can systematically meet three major needs: in terms of autonomous needs, the calling drives ideological and political theory teachers in colleges and universities to regard teaching and scientific research as a choice of self-realization, rather than a task or responsibility (Bunderson and Thompson, 2009). This internal drive directly strengthens the internalization of teachers’ work objectives, that is, organizational identification, which is in line with the logic of S-DT about the satisfaction of independent needs → the consistency of behavior and values (Van den Broeck et al., 2016). As far as competency needs are concerned, the sense of calling urges teachers to take the initiative to invest resources, such as learning new methods of ideological and political education, participating in teaching reform, and enhancing their confidence in their own abilities in the positive feedback of completing tasks-obtaining growth. This ability matching and growth experience further enhances teachers’ willingness to contribute to the organization, that is, participation (Gagné and Deci, 2005). As far as the need for belonging is concerned, calling strengthens the emotional connection between teachers and organizations through the perception of the fitness between work meaning and organizational goals. This kind of identity directly enhances the persistence tendency of teachers to maintain the membership of the organization, that is, loyalty (Dobrow and Tosti-Kharas, 2011). Empirical studies have also shown that calling can significantly improve the level of organizational commitment by meeting the individual’s autonomy, competence and belonging needs (Lobene and Meade, 2013; DiRenzo et al., 2022; Park and Hai, 2024).

Further, organizational commitment, as a bridge between individual and organization (Allen and Meyer, 1990), will strengthen work engagement through the path of emotional attachment to resource input, so that ideological and political theory teachers in colleges and universities will show more positive classroom interaction, more in-depth scientific research exploration and other behaviors (Riketta, 2008). If addition, SET can further complement this process: if individuals identify with and rely on the organization and are grateful for everything given by the organization, they will return to the organization and actively participate in various tasks delivered by the organization (Sezen-Gultekin et al., 2021). On the other hand, due to the fear of losing all kinds of favorable conditions in the organization, individuals will also strive to integrate into their own work roles and complete the work delivered by the organization as well as possible (Zhou and Pan, 2011). Based on the above analysis, this study proposes the hypothesis

Hypothesis (H2): Organizational commitment plays a mediating role between calling and work engagement of ideological and political theory teachers in colleges and universities.

### 2.3 The mediating role of job satisfaction

Job satisfaction refers to a multi-dimensional and context-dependent positive psychological state that arises when individuals perceive that their work-related needs, expectations, and values are fulfilled through the cognitive and emotional evaluation of various job-related elements, including job characteristics (e.g., task significance, work autonomy), organizational environment (e.g., interpersonal support, reward fairness), and career development opportunities (Locke, 1976; Aziri, 2011; Judge et al., 2020). This state is not only a simple emotional experience of happiness but also involves a comprehensive cognitive judgment of the congruence between work outcomes and personal aspirations (Judge et al., 2001). Career calling reflects the individual’s strong desire and passion for the specific work they should be engaged in, indicating that individuals with a sense of calling are mainly affected by endogenous motivation in career decision-making (Duffy et al., 2018). From the perspective of incentive efficiency, this endogenous driving force can continuously stimulate the goal-oriented behavior and self-incentive mechanism of ideological and political theory teachers in colleges and universities, which not only helps to improve the quality of the completion of work tasks, but also effectively enhances the pleasant experience of work through the achievement feedback cycle (Duffy et al., 2014; Zhang and Hirschi, 2021; Schultz et al., 2022).

SET provides a key perspective for understanding the mechanism of job satisfaction and work engagement (Cropanzano and Mitchell, 2005). The theory holds that the behavior of individuals in the organization is essentially a kind of reciprocal exchange: when individuals perceive that the organization provides resources, such as emotional support, career development opportunities, or recognition, such as affirmation of work value, that exceed expectations, they will have a psychological drive of reward obligation (Garg et al., 2018; Zang and Feng, 2023). Specific to the ideological and political theory teachers in colleges and universities, job satisfaction, as a manifestation of positive emotional state, will strengthen teachers’ reward motivation (Kašpárková et al., 2018; Topchyan and Woehler, 2021), that is, in order to maintain a benign exchange relationship with the organization, teachers will be more active in their work, such as increasing lesson preparation time and participating in teaching reform (Karanika-Murray et al., 2015), so as to further strengthen the support and recognition of the organization through higher work engagement (Urbini et al., 2020; Yildiz and Yildiz, 2022). Based on the above analysis, this study posits the following hypothesis:

Hypothesis (H3): Job satisfaction plays a mediating role between calling and work engagement of ideological and political theory teachers in colleges and universities.

### 2.4 The chain mediating effect of organizational commitment and job satisfaction

Based on the above analysis, organizational commitment and job satisfaction play a mediating role between career calling and work engagement of ideological and political theory teachers in colleges and universities. According to the theoretical hypothesis of the multiple mediating model, whether there is interaction between multiple mediating variables determines the type of the multiple mediating model (Hayes, 2009). Specifically, if there is no interaction between multiple mediation variables, the model is a parallel multiple mediation model; if there is mutual influence and shows sequential characteristics to form a mediation chain, the model is a chained multiple mediation model (Fang et al., 2014). Therefore, whether organizational commitment and job satisfaction play a parallel mediating or chain mediating role between calling and work engagement of ideological and political theory teachers in colleges and universities depends on the influence relationship between organizational commitment and job satisfaction.

According to the Social Information Processing Model, individuals form psychological and behavioral responses through the path of situational information input to cognitive processing to attitude/behavior output (Salancik and Pfeffer, 1978). In this process, contextualized organizational information as a pre-input, through the individual’s cognitive reconstruction, ultimately affect their attitudes and behaviors (Wilson and Gilbert, 2005). Organizational commitment, as a kind of cognition of macro-organizational ecology, will affect individuals’ experience of micro work field (Cui et al., 2012). Teachers with high organizational commitment will pay more attention to “meaning clues” in their work, such as the growth of students’ values and the social value of ideological and political theory courses, rather than only “task indicators” such as class hours and scientific research pressure; this kind of “meaning focus” will enhance their emotional engagement in work, and then improve job satisfaction (Abu Orabi et al., 2024). Further, the calling of ideological and political theory teachers in colleges and universities can promote their organizational commitment; organizational commitment, as a pre-contextual cognition, affects job satisfaction by reconstructing individuals’ perception of the meaning of work. As a result of needs satisfaction, job satisfaction further drives work engagement, which constitutes a chain intermediary path of calling to organizational commitment to job satisfaction to work engagement. Therefore, this study proposes a hypothesis:

Hypothesis (H4): Organizational commitment and job satisfaction play a chain mediating role between calling and work engagement of ideological and political theory teachers in colleges and universities.

**Figure 1:**
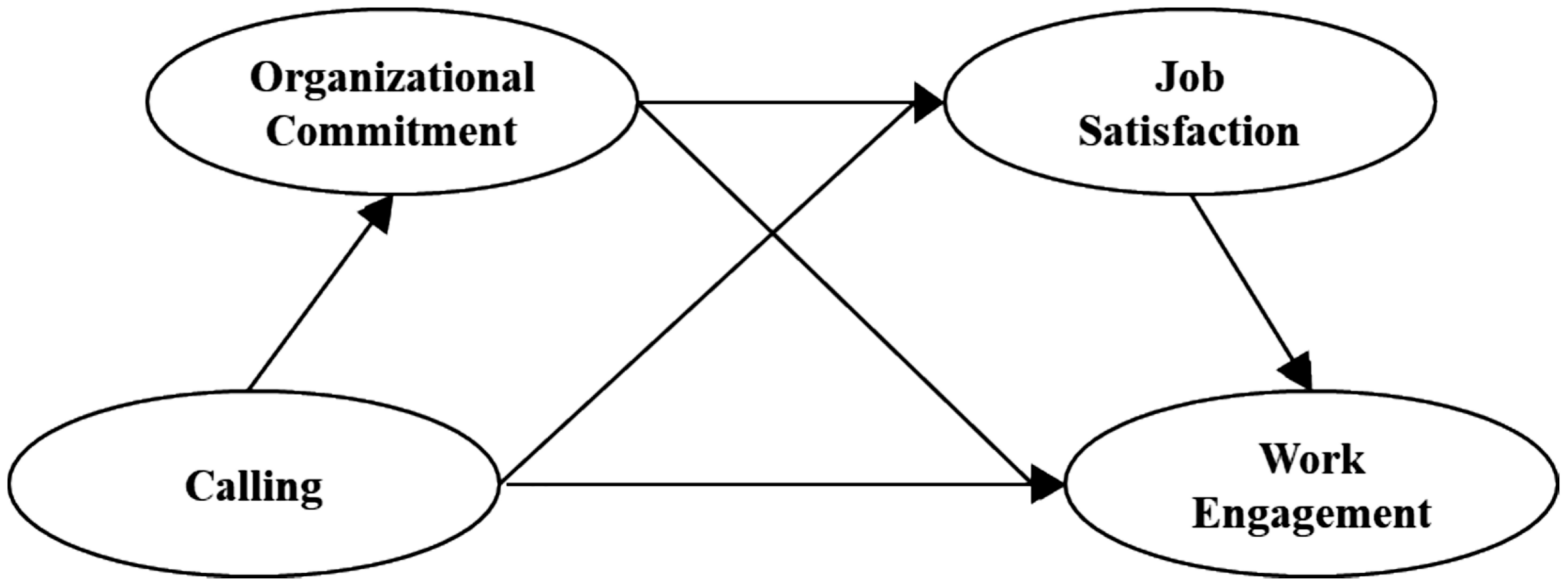
The proposed moderated mediation model.

Based on the above analysis, this study establishes a chain mediating model with the independent variable as calling, the dependent variable as work engagement, and the mediating variable as organizational commitment and job satisfaction, to reveal the mechanism of calling on the work engagement of ideological and political theory teachers in colleges and universities, as shown in Figure 1.

## 3 Materials and methods

### 3.1 Participants and procedure

This study used the “snowball” method to issue electronic questionnaires, that is, to issue questionnaires to ideological and political theory teachers of colleges and universities in the researcher ‘s social network through the questionnaire star platform, and then entrust them to issue questionnaires to other teachers as much as possible, so as to continuously roll the snowball. After 1 month, 1893 questionnaires were collected in this study, and the samples were from different types of universities in 13 provinces, autonomous regions and municipalities in China. After eliminating invalid questionnaires such as too short answer time (≤100 s), a total of 1,428 valid questionnaires were obtained, with an effective recovery rate of 75.4%.

Based on previous research (Li and Wang, 2022; Shang, 2022; Cui and Yin, 2023; Wang et al., 2024), this study examined participants’ gender, age, educational background, professional title, and the type of institution where they teach. The specific composition of the sample is as follows: There were 355 males (24.9%) and 1,073 females (75.1%). There were 304 people aged 35 and below (21.3%), 494 people aged 36–45 (34.6%), 368 people aged 46–55 (25.8%), and 262 people aged 56 and above (18.3%); 278 (19.5%) had bachelor’s degree or below, 831 (58.2%) had master‘s degree, and 319 (22.3%) had doctor‘s degree. There were 169 assistant professors (11.8%), 632 lecturers (44.3%), 444 associate professors (31.1%) and 183 professors (12.8%). There were 443 people (31.0%) in advance professional school, 95 people (6.7%) in junior colleges, 620 people (43.4%) in ordinary undergraduate colleges, and 270 people (18.9%) in double first-class construction colleges. In general, the demographic characteristics of the sample personnel are basically in line with the actual situation of ideological and political theory teachers in Chinese colleges and universities.

### 3.2 Measures

All scales used in this study were translated into Chinese (for scales originally developed in English) or directly adopted Chinese versions (for scales originally compiled in Chinese), and were distributed to participants in Chinese to ensure accurate understanding and effective response.

#### 3.2.1 Calling scale

The scale compiled by Zhang (2015) was used to measure the sense of calling. The scale includes three dimensions: orientation, altruistic contribution and initiative, with a total of 10 items. The sample item is as follows: “Compared with other occupations, I think I should be engaged in the current occupation.” Answer options range from 1 (completely inconsistent) to 7 (completely consistent), and the higher the score, the higher the individual’s sense of calling. The Cronbach’s *α* value of the scale in this study was 0.952.

#### 3.2.2 Organizational commitment scale

Organizational commitment adopts the scale compiled by Chen et al. (2006). The scale has 8 items. The sample item is as follows: “I have a strong sense of belonging to the company.” The answer options range from 1 (completely inconsistent) to 7 (completely consistent), and the higher the score, the higher the individual‘s organizational commitment level. The Cronbach‘s α value was 0.905.

#### 3.2.3 Job satisfaction scale

Job satisfaction was measured by the scale compiled by Cui et al. (2012). The scale contains two dimensions, external satisfaction and internal satisfaction, a total of 6 items. The sample item is as follows: “Are you satisfied with the current working environment.” Answer options range from 1 (completely dissatisfied) to 7 (completely satisfied). The higher the score, the higher the individual‘s satisfaction with the job. The Cronbach‘s α value was 0.834.

#### 3.2.4 Work engagement scale

The work engagement scale was developed by Schaufeli et al. (2002a) and Schaufeli et al. (2002b) and revised by Zhang and Gan (2005). This scale has been proved to have good reliability in the context of Chinese culture. The scale includes three dimensions: vigor, dedication and absorption, with a total of 15 items. The sample item is as follows: “When I work, time always goes fast.” Answer options range from 1 (never) to 7 (always). The higher the score, the higher the individual‘s work engagement. The Cronbach‘s α value was 0.874.

### 3.3 Data analysis

In this study, SPSS 26.0 was used to test the artificial covariation between the predictive variables and the criterion variables, as well as descriptive statistics and correlation analysis. The discriminant validity test of the main variables was carried out by Mplus 8.3 and the structural equation model was established to explore the predictive effect of the sense of calling on the work engagement of ideological and political theory teachers in colleges and universities, as well as the chain mediating effect of organizational commitment and job satisfaction between them, and the significance of the mediating effect was tested according to Bootstrap.

## 4 Result

### 4.1 Common-method bias test

According to the program control and statistical control methods (Zhou and Long, 2004), this study controls and tests the common-method bias through the following two steps: First, it adopts the anonymous filling method, and emphasizes the anonymity, confidentiality, and no right and wrong answers of the survey through the instruction in the data collection process. And the data is only for academic research, reducing the respondents’ vigilance and enhancing the willingness to respond truthfully. The second is to conduct a statistical test on the degree of common-method bias of this study. The results of Harman single factor analysis of variance showed that four factors with eigenvalues greater than 1 were extracted from the results of non-rotating exploratory factor analysis, and the maximum factor variance interpretation rate was 31.0%, less than 40%. Although the Harman single factor test cannot fully reflect the potential method bias (Podsakoff et al., 2024), the method still has certain indicative value. The statistical results show that the maximum factor interpretation rate does not reach the traditional threshold (40%), indicating that there is no significant evidence that there is serious artificial co-variation between the predictor variable and the criterion variable.

### 4.2 Discrimination validity test of variables

In this study, the confirmatory factor analysis method was used to test the discriminant validity of the main variables, and the results were shown in Table 1. The *X*^2^/df value of the benchmark model (four-factor model) was 1.127, RMSEA was 0.009, CFI was 0.997, TLI was 0.997, SRMR was 0.020, and each fitting index met the standard. At the same time, compared with other alternative models, the benchmark model constructed in this study has the best fit. There is a good discriminant validity between the variables in this study.

**Table 1:** Variable discrimination validity test results.

Model	*X* ^2^	df	RMSEA	CFI	TLI	SRMR
Benchmark model	784.377	696	0.009	0.997	0.997	0.020
Three-factor model	6573.411	699	0.077	0.785	0.772	0.112
Two-factor model	7869.469	701	0.085	0.738	0.723	0.116
Single factor model	10887.338	702	0.101	0.627	0.607	0.121

The benchmark model includes career calling, organizational commitment, job satisfaction and work engagement; the three-factor model is career calling + organizational commitment, job satisfaction, and work engagement; the two-factor model is career calling + organizational commitment, job satisfaction + work engagement; the single factor model is career calling + organizational commitment + job satisfaction + work engagement.

### 4.3 Descriptive statistics and correlation matrix

Table 2 shows the mean, standard deviation and correlation coefficient of the main variables in this study. The results show that there is a significant positive correlation between calling, organizational commitment, job satisfaction and work engagement of ideological and political theory teachers in colleges and universities, which preliminarily supports the hypothesis of this study.

**Table 2:** Descriptive statistics and correlation matrix results.

Variable	*M*	SD	Calling	OC	JS	WE
Calling	3.488	1.167	–			
OC	4.311	0.905	0.177^**^	–		
JS	3.706	0.813	0.502^**^	0.233^**^	–	
WE	3.901	0.648	0.565^**^	0.439^**^	0.542^**^	–

*p < 0.05, **p < 0.01, ***p < 0.001; OC, organizational commitment; JS, job satisfaction; WE, work engagement.

### 4.4 Chain mediating effect analysis

This study uses structural equation model to explore the relationship between career calling, organizational commitment, job satisfaction and work engagement of ideological and political theory teachers in colleges and universities, and uses Bootstrap method to test the significance of mediating effect. In Mplus 8.3, 5,000 Bootstrap samples are set to be extracted. If the Bootstrap 95% confidence interval does not contain 0, the parameter estimates are significant.

**Figure 2:**
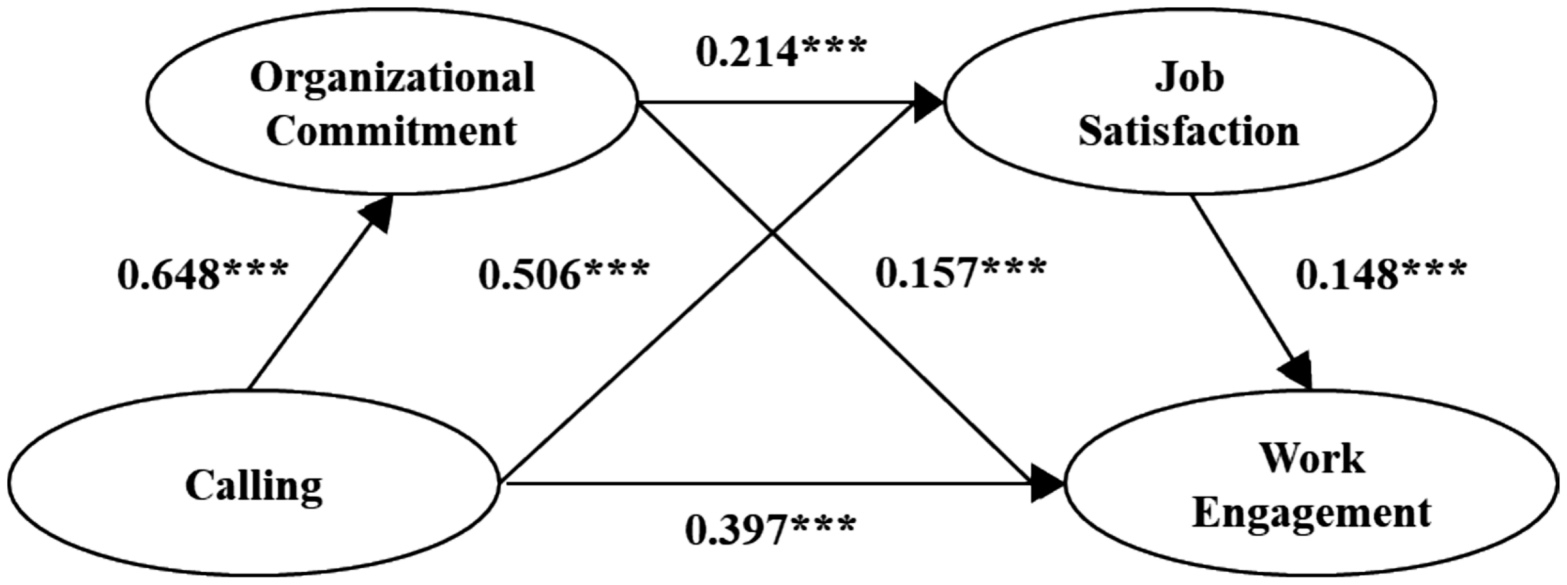
Structural equation model results.

The results of the model operation (standardized coefficient) are shown in Figure 2. The calling can significantly positively predict organizational commitment (*β* = 0.648, *p* < 0.001). Calling and organizational commitment could significantly and positively predict job satisfaction (*β* = 0.506, *p* < 0.001; *β* = 0.214, *p* < 0.001); calling, organizational commitment and job satisfaction all had a significant positive impact on work engagement (*β* = 0.397, *p* < 0.001; *β* = 0.157, *p* < 0.001; *β* = 0.148, *p* < 0.001).

The mediating effect Bootstrap analysis results are as shown in Table 3. The direct effect of career calling on work engagement is 0.397, and the corresponding confidence interval is [0.338,0.455], excluding 0, indicating that H1 is supported. Among the three mediating effect paths, the effect value of path 1 calling to organizational commitment to work engagement is 0.102, and the confidence interval is [0.067,0.137]; the effect value of path 2 calling to job satisfaction to work engagement is 0.075, confidence interval was [0.043,0.105]; the effect value of path 3 calling to organizational commitment to job satisfaction to work engagement is 0.021, and the confidence interval is [0.011,0.032]. The confidence intervals of the three mediating effect path coefficients do not include 0, indicating that the mediating effect is significant, and H2,3,4 are supported.

**Table 3:** Bootstrap analysis of mediating effect of chain mediation model.

Effect path	Effect value	Standard error	Boot CI 95%	
			Lower limit	Upper limit
Total effect	0.595	0.015	0.564	0.624
Direct effect	0.397	0.030	0.338	0.455
Total indirect effect	0.198	0.026	0.144	0.247
Path 1: calling → OC → WE	0.102	0.018	0.067	0.137
Path 2: calling → JS → WE	0.075	0.016	0.043	0.105
Path 3: calling → OC → JS → WE	0.021	0.005	0.011	0.032

Further analysis from Table 3 shows that the direct effect, path 1, path 2 and path 3 account for 33.2,17.1, 12.6 and 3.5% of the total effect of the model, respectively. Comparing the three paths, the first is that the effect value of path 1 (calling to job satisfaction to work engagement) is the largest, followed by path 2 (calling to organizational commitment to work engagement), and finally path 3 (calling to organizational commitment to job satisfaction to work engagement). Moreover, the effect value of path 1 was significantly higher than that of path 3 (*p* < 0.001), the effect value of path 2 was significantly higher than that of path 3 (*p* < 0.001), the difference between path 1 and path 3 is not significant (*p* > 0.05).

## 5 Discussion

Based on S-D T, SET, SIP Model and previous empirical research, this study constructs a chain mediating model to examine the specific mechanism of career calling on the work engagement of ideological and political theory teachers in colleges and universities.

The results showed that career calling has a significant positive effect on the work engagement of ideological and political theory teachers in colleges and universities, which supports hypothesis 1. This finding shows that ideological and political theory teachers with a sense of calling will regard the meaning of work as the meaning of their own life, so as to be more engaged in the work of ideological and political education driven by their own inner will. The results contribute to the existing literature by revealing the direct effect of calling on the work engagement of ideological and political theory teachers in colleges and universities, and expand the academic community’s understanding of career calling and work engagement of ideological and political theory teachers in colleges and universities. Moreover, this research finding is consistent with the previous empirical research results in other groups (Zhang et al., 2024), which once again confirms the important influence of the sense of calling on work engagement, and shows that this influence is not only applicable to groups such as enterprise employees, but also to ideological and political theory teachers in colleges and universities, which has the effectiveness of crossing different groups. In addition, the results of this study further elaborate and verify the core framework of S-DT, which posits that the satisfaction of three basic psychological needs, that is, autonomy, competence, and relatedness, are key drivers of individual well-being and proactive behaviors (Deci and Ryan, 2012). Specifically, career calling enhances autonomy for ideological and political theory teachers by aligning their work with inner values and life meaning, enabling them to engage in teaching and education driven by intrinsic motivation rather than external demands. Beyond autonomy, calling also fosters competence: the transcendent mission of ideological and political education (e.g., cultivating socialist successors) motivates teachers to continuously improve their theoretical literacy and teaching skills, and the positive feedback from students’ value shaping further reinforces their sense of professional competence. Additionally, calling strengthens relatedness: ideological and political teachers with a strong sense of calling tend to perceive their work as connecting individual efforts to national development and student growth, enhancing their sense of connection with the organization, colleagues, and students. The comprehensive satisfaction of these three basic psychological needs ultimately promotes work engagement, which not only confirms the hypothesis that the autonomy of behavioral motivation positively predicts individual well-being (Conway et al., 2015) but also fully embodies the integrated explanatory power of S-DT, thereby enhancing the universality of this theory in the context of ideological and political education.

The study found that organizational commitment has a mediating effect between the sense of calling and work engagement of ideological and political theory teachers in colleges and universities, which supports hypothesis 2. This shows that the sense of calling can stimulate the strengthening effect of ideological and political theory teachers’ professional role identity, and then form a continuous tendency of organizational attachment, and ultimately improve the level of their work engagement. This result not only clarifies the mediating mechanism between career calling and work engagement in the specific group of college ideological and political theory teachers, but also extends the application of organizational commitment as a mediator in the relationship between calling and work engagement to the context of college ideological and political education, which opens up new ideas for understanding the mechanism between calling and work engagement. In addition, this result also confirms the S-DT framework of basic psychological needs (Deci and Ryan, 2000), and the inference of SET that individual identity and dependence on the organization will reward the organization through active work (Zhou and Pan, 2011).

The study found that job satisfaction has a mediating effect between the sense of calling and work engagement of ideological and political theory teachers in colleges and universities, which supports hypothesis 3. This shows that the sense of calling, as an endogenous driving force of work, can effectively enhance job satisfaction through the achievement feedback cycle, and ultimately help to improve the work engagement of ideological and political theory teachers in colleges and universities. This result also elaborates on the mediating mechanism of job satisfaction between calling and work engagement in the group of college ideological and political theory teachers, and verifies the applicability of job satisfaction as a mediator in the calling-work engagement relationship to this specific professional group. In addition, this result also confirms the view of SET that the behavior of individuals in the organization is based on a reciprocal relationship (Cropanzano and Mitchell, 2005).

The study also found that organizational commitment and job satisfaction have a chain mediating effect between the sense of calling and work engagement of ideological and political theory teachers in colleges and universities, which supports hypothesis 4. This shows that the sense of calling can affect the identity, participation and loyalty of college ideological and political theory teachers to the organization, and then affect their impression and evaluation of the work, and ultimately affect their engagement in ideological and political education. This result further deepens the understanding of the multi-stage mediating mechanism between calling and work engagement among college ideological and political theory teachers, and confirms the chain mediating role of organizational commitment and job satisfaction in this specific group. It also confirms the view of the SIP Model, that is, the input information of the situation can affect the adjustment of individual attitudes and beliefs (Salancik and Pfeffer, 1978).

## 6 Practical implications and limitations

Based on the above research conclusions, the four measures of value guidance, organizational reengineering, dynamic activation and dynamic regulation can help to improve the level of work engagement of ideological and political theory teachers in colleges and universities.

First, build a career calling cultivation system and strengthen the guidance of intrinsic value. In the process of talent selection, relevant managers should introduce career calling evaluation tools, incorporate professional value identification into the post competency model of ideological and political theory teachers in colleges and universities, establish a morality-oriented admission mechanism (i.e., a selection criterion that prioritizes teachers’ moral qualities and educational dedication), and screen out candidates with strong educational feelings. At the level of teacher training, based on the platforms of famous teacher studio of ideological and political courses in colleges and universities and national training base of ideological and political theory teachers in colleges and universities, managers can deepen the recognition of professional value of ideological and political theory teachers in colleges and universities and strengthen the internal foundation of work input through cognitive strengthening means such as theoretical study, practical understanding and model demonstration.

Second, optimize the organizational support system and improve the level of organizational commitment. In terms of institutional support, the manager should formulate the “Regulations on the Guarantee of Professional Development of Ideological and Political Theory Teachers in Colleges and Universities,” and clarify the institutional guarantees such as special allowances, academic leave and mental health services; at the level of instrumental support, managers should establish a “double-qualified” tutorial system, equip young teachers with double tutors for teaching and scientific research, and develop an OBE (Outcome Based Education) ability development platform exclusive to ideological and political theory teachers in colleges and universities. In the dimension of emotional support, managers should implement the “Organizational Communication Quality Improvement Plan,” that is, through regular informal organizational activities such as leader’s talk days and interdisciplinary teaching and research salons, teachers’ sense of identity and belonging to the organization should be enhanced, so as to improve their level of work engagement.

Third, improve the job satisfaction drive system and stimulate endogenous motivation. In terms of support mechanism, managers can build a “four-dimensional resource support system for ideological and political theory teachers in colleges and universities”: add ideological and political special teaching assistant posts (human resources), develop virtual simulation teaching resource base (technical resources), set up classroom innovation seed fund (financial resources), and establish an inter-school teaching and research community (social resources). In terms of workload, a workload dynamic monitoring system can be established to strictly control the teacher-student ratio within 1: 350, ensure a reasonable workload, and achieve the gain effect of job satisfaction on work engagement.

Fourth, build a dynamic monitoring mechanism to achieve precise empowerment. Managers should establish a functional monitoring platform for the work engagement level of college ideological and political theory teachers, and monitor the dynamic changes of teachers’ career calling, organizational commitment, job satisfaction, and work engagement through a big data-based accurate portrait system. On this basis, develop an intelligent early warning system: first, clarify the core monitoring dimensions (i.e., the four variables mentioned above) and set scientific thresholds for each dimension by referring to industry benchmarks, regional average levels of the same group, and the institution’s own development goals; second, define the corresponding intervention mechanisms for different early warning levels (e.g., one-on-one mentoring for low career calling, professional training support for low job satisfaction, and organizational care interviews for low organizational commitment). This system will automatically trigger the matched intervention mechanism when any dimension index is lower than the preset threshold. Additionally, establish a closed-loop mechanism of “problem diagnosis - result feedback - targeted improvement” (specifically, this loop connects monitoring results with follow-up interventions and resource allocation: first, diagnose the key factors affecting teachers’ work engagement through monitoring data; then, feed back the diagnosis results to managers and teachers; finally, formulate and implement targeted improvement measures, and re-evaluate the effect through the monitoring platform to form continuous optimization), link the monitoring results with resource allocation (e.g., training resources, incentive funds, and career development opportunities), and form a mechanism of precise empowerment and continuous improvement. This will help effectively improve the work engagement level of college ideological and political theory teachers, thereby contributing to the implementation of the fundamental task of moral education in colleges and universities.

Although this study puts forward a series of valuable conclusions, there are still some limitations:

First, despite procedural control and statistical tests for common method bias (no serious artificial covariation confirmed), social desirability may still lead to residual bias. Future research can adopt multi-method triangulation to make up for this: (1) collecting objective behavioral data (e.g., classroom interaction duration, research output, student feedback) to compare with self-reported work engagement; (2) using accessible wearable devices to track physiological indicators (e.g., heart rate variability, sleep quality) associated with work engagement; (3) conducting semi-structured interviews to supplement qualitative explanations. Priority can be given to combining objective behavioral data and interviews (more operable for most institutions), with physiological measurement selected flexibly based on research resources.

Second, this study adopts a cross-sectional design, which cannot determine the causal relationship or dynamic influence mechanism between variables (e.g., time sequence, reverse causality). Future research can use a 3–5-year longitudinal tracking design with cross-lagged structural equation modeling to test the temporal effect of variables, and introduce fixed-effects models to control time-invariant individual characteristics, thereby enhancing causal inference.

Third, there is gender bias in the sample composition (females: 75.1%, males: 24.9%), which deviates from the overall distribution of college ideological and political theory teachers. This imbalance may omit the interaction between gender and core variables and weaken the external validity of the conclusions. Future research can improve the universality of results through gender-matched stratified sampling or male teacher subgroup analysis.

Fourth, the snowball sampling method may lead to sample homogeneity bias, limiting representativeness. Although invalid questionnaires were excluded and common method bias was controlled, the inherent limitations of this sampling method affect the extrapolation of conclusions. Future research can combine stratified random sampling (stratified by university type, region, teaching experience) or cluster sampling to enhance sample diversity.

Fifth, this study only examines the mediating roles of organizational commitment and job satisfaction, without including potential confounding variables (e.g., workload, contract type, institutional resource support, teaching experience) that may affect both career calling and work engagement. These variables may limit the comprehensive understanding of the core relationship. Future research should incorporate these variables to control their effects or test their mediating/moderating roles, so as to fully reveal the driving path of career calling on work engagement.

## Data Availability

The datasets presented in this article are not readily available because thank you for your inquiry regarding the data restrictions applicable to our dataset. We appreciate the journal’s commitment to transparency and responsible research practices, and we would like to clarify our approach to data sharing in this context. Our study focuses on a specific professional group—college ideological and political course teachers—and involves sensitive details about their career calling, organizational commitment, and job satisfaction. During data collection, we obtained formal approval from the institutional review board (IRB) of Harbin Engineering University, which required strict adherence to ethical guidelines, including the protection of participants’ privacy and confidentiality. Specifically, the dataset contains identifiable contextual information (e.g., institutional affiliations, job titles, and nuanced qualitative reflections) that, if disclosed publicly, could potentially trace back to individual respondents, even after de-identification efforts. Given the small sample size and the specialized nature of the population, we believe maintaining anonymity is critical to upholding their trust and ensuring the integrity of future research involving similar groups. Additionally, the dataset is deeply embedded in the unique cultural and institutional context of Chinese higher education, where the roles and challenges of ideological and political course teachers are shaped by specific policy environments and professional norms. We have structured the analysis to prioritize interpretability within this context, and we are cautious about the risk of misinterpretation if the data were to be analyzed outside its original framework without our guidance. That said, we remain committed to academic openness and are happy to explore reasonable requests for data access under controlled conditions. For example, we would consider sharing anonymized subsets of the dataset (e.g., aggregated quantitative variables or redacted qualitative excerpts) to support independent verification of key findings, provided that such sharing does not compromise participant confidentiality. Please let us know if the journal requires specific documentation (e.g., an IRB approval letter) to confirm our compliance with ethical standards, and we will provide it promptly. We hope this explanation aligns with the journal’s policies, and we remain confident that our study makes a valuable contribution to understanding calling and work engagement in higher education. Thank you again for your understanding. Requests to access the datasets should be directed to zms@nefu.edu.cn.
